# Comparative efficacy of free and PNAGA-encapsulated antimalarial drugs: temperature-dependent modulation of *Plasmodium falciparum* growth

**DOI:** 10.3389/fphar.2026.1767493

**Published:** 2026-08-20

**Authors:** Daniel Mutangadura, Paul Mushonga, Takafira Mduluza

**Affiliations:** 1 Department of Biotechnology and Biochemistry, University of Zimbabwe, Harare, Zimbabwe; 2 Department of Chemistry and Earth Sciences, University of Zimbabwe, Harare, Zimbabwe

**Keywords:** controlled drug release, mefloquine hydrochloride, Plasmodium falciparum, PNAGA, pyrimethamine, temperature-responsive (UCST) polymer, thermosensitive hydrogel

## Abstract

**Introduction:**

Poly(N-acryloyl glycinamide) (PNAGA) was evaluated as a temperature-responsive carrier for antimalarial drugs.

**Methods:**

PNAGA was synthesized and characterized by DSC and TGA, and its sol–gel transition was determined by the reverse test-tube method. Encapsulation efficiency (EE%) of mefloquine hydrochloride and pyrimethamine in PNAGA was determined by UV-Vis spectrophotometry, using dialysis to separate free from encapsulated drug and calibration curves at each drug's characteristic absorbance maximum (284 nm for mefloquine hydrochloride, 272 nm for pyrimethamine). Free drugs and PNAGA-encapsulated formulations were introduced to Plasmodium falciparum cultures, alongside PNAGA alone and a negative control, and parasitemia was assessed after 24 h at 37 °C and again after 24 h at 4 °C.

**Results:**

DSC and TGA analyses demonstrated PNAGA's amorphous, thermoreversible nature and established its thermal stability limits. The sol–gel transition was 22.6 °C ± 1 °C, confirming UCST behavior. PNAGA efficiently encapsulated mefloquine hydrochloride and pyrimethamine, with encapsulation efficiencies of 93.3% and 94.7%, respectively. After 24 h at 37 °C, parasitemia was significantly lower in cultures treated with free drugs than with polymer-bound formulations, indicating that encapsulation delayed drug release and reduced immediate efficacy. PNAGA alone showed no inhibitory effect. After 24 h at 4 °C (below the sol–gel transition), PNAGA–mefloquine matched free mefloquine in efficacy, while PNAGA–pyrimethamine remained less effective than free pyrimethamine.

**Discussion:**

These findings confirm that PNAGA is a reproducible, temperature-responsive hydrogel that efficiently encapsulates antimalarial drugs and modulates their availability according to its UCST, with reduced immediate efficacy at 37 °C (above the sol–gel transition) and higher efficacy below the sol–gel transition (4 °C) for PNAGA–mefloquine.

## 1 Introduction

Malaria, a disease transmitted through mosquito bites, is caused by *Plasmodium* parasites and remains a significant public health concern globally. In 2021 alone, the World Health Organization recorded 241 million cases, resulting in 630,000 deaths, primarily in sub-Saharan Africa (WHO, 2022). Among the different *Plasmodium* species, *Plasmodium falciparum* is the most lethal, often leading to severe complications like cerebral malaria (Schiess et al., 2020).

The life cycle of *Plasmodium* involves a complex interaction between humans and female Anopheles mosquitoes. When an infected mosquito bites a human, it injects sporozoites into the bloodstream, which then reach the liver to mature and multiply. These parasites transform into merozoites, invading red blood cells to replicate further. The cycle continues as infected cells burst, releasing new merozoites. Some of these merozoites differentiate into sexual gametocytes, which mosquitoes ingest during feeding (Alonso et al., 2011). Within the mosquito, the gametocytes develop into sporozoites, ready to infect a new human host. Malaria typically manifests with recurring fever, chills, sweating, and headaches. However, severe cases can escalate into life-threatening conditions like severe anemia, acute respiratory distress syndrome (ARDS), and cerebral malaria (World Health Organization, 2010).

Drug resistance in *Plasmodium* parasites, particularly to artemisinin derivatives in Southeast Asia, is a significant obstacle to malaria control. The development of new antimalarial drugs with unique mechanisms of action is crucial for several reasons. Firstly, novel drugs are needed to address existing and emerging drug resistance, ensuring the continued effectiveness of malaria treatment (Ashley et al., 2014). Secondly, current antimalarial drugs often have limitations such as short half-lives, complex administration regimens, and limited effectiveness against certain *Plasmodium* species, which new drugs could potentially overcome (Ashley et al., 2014). Finally, targeting various stages of the parasite’s intricate life cycle could offer broader-spectrum activity against diverse *Plasmodium* species, potentially disrupting transmission and advancing malaria elimination efforts (Singh and Rathi, 2023).

In recent years, the incorporation of malaria drugs into polymers and nanoparticles has been the subject of many studies. Primaquine, an antimalarial drug with high dosage requirements, was loaded onto poly (lactic-co-glycolic) acid nanoparticles, and the formulation was orally administered to mice. The nanoformulation was 20% more effective than the standard oral dose (Bhargava et al., 2024). In another study, an atovaquone solid drug nanoparticle injectable formulation was shown to have a longer-acting prophylaxis against *Plasmodium berghei* ANKA malaria in C57BL/6 mice than free atovaquone (Bakshi et al., 2018). The studies demonstrate the potential of drug delivery vehicles in enhancing the efficacy of malaria drugs.

A study explored the use of polymeric nanoparticles for delivering atovaquone, an antimalarial drug targeting the mitochondria of *Plasmodium*, demonstrating enhanced solubility, stability, and oral bioavailability, which led to improved therapeutic efficacy in malaria-infected mice (Borhade et al., 2014). These findings underscore the potential of polymeric drug delivery systems (DDS) to optimize atovaquone’s efficacy, particularly given its transmission-blocking capabilities. The expected enhancements through polymeric DDS could be leveraged to strengthen malaria prevention strategies.

Poly (N-acryloyl glycinamide) (PNAGA) hydrogels, a class of polymers that are sensitive to temperature changes, are being investigated as a promising drug delivery platform. Their distinctive characteristic is the ability to transform from a liquid state into a gel when exposed to changing temperatures. This shift is triggered by reaching the polymer’s critical solution temperature (Xu and Liu, 2018).

The thermo-responsive nature offers unique advantages for drug delivery. Drugs are easily incorporated into the liquid polymer solution at lower temperatures. Upon administration, the body’s warmth triggers the gelation process at the target site, trapping the drug within the hydrogel network. This *in situ* gel formation ensures minimally invasive drug delivery and a controlled, sustained release, enhancing therapeutic effectiveness (Klouda and Mikos, 2008).

PNAGA hydrogels possess several ideal features for drug delivery. Notably, they are biocompatible and show low toxicity, ensuring safety within the body (Majstorović et al., 2023). Furthermore, the critical solution temperature can be adjusted by modifying the polymer composition, allowing for a tailored drug release rate to match specific therapeutic needs (Zhou et al., 2023). As a biodegradable material, these hydrogels naturally break down and are removed from the body over time, minimizing any potential adverse effects (Musuc and Chelu, 2024).

Thermosensitive hydrogels have demonstrated their adaptability by enveloping a wide range of therapeutic agents, including small-molecule drugs, proteins, and nucleic acids (Van Tomme et al., 2008). This flexibility enables the delivery of various drugs for different treatment purposes. Additionally, the hydrogel network shields encapsulated drugs from degradation, preserving their stability and efficacy until released (Bromberg and Ron, 1998).

In recent years, thermosensitive hydrogels have drawn considerable attention as a promising channel for antimalarial drug delivery. These polymers, which transition from a liquid to a gel state in response to temperature changes, offer unique advantages in controlling drug release and improving therapeutic outcomes. A thermosensitive hydrogel developed for the sustained release of artemisinin exhibited excellent biocompatibility and maintained drug release for over 2 weeks, significantly reducing parasite populations within a mouse model infected with malaria (Li et al., 2020).

Drug delivery systems (DDS) offer a promising avenue in combating malaria, especially within transmission-blocking strategies. By tailoring drug release and targeting specific parasite life cycle stages, DDS can significantly enhance the efficacy and safety of antimalarial drugs, thereby diminishing transmission risks. Liposomes, spherical vesicles formed by phospholipid bilayers, have been extensively researched as drug delivery systems (DDS) for antimalarial drugs. Their capacity to encapsulate both hydrophilic and hydrophobic drugs, combined with their biocompatibility and biodegradability, makes them an ideal platform for targeted drug delivery. A notable study explored the use of liposomes for delivering primaquine, an antimalarial drug targeting the sexual stages of the malaria parasite, demonstrating improved bioavailability while reducing toxicity, resulting in a more effective transmission-blocking effect in mice (Singh and Vingkar, 2008).

Mefloquine remains a clinically effective antimalarial drug with a 2–4-week half-life, recommended weekly by WHO and CDC for prophylaxis in travellers (Ahmad et al., 2021; Tickell-Painter et al., 2017). A Cochrane review judged it efficacious for preventing malaria in pregnancy across sub-Saharan Africa, though worse-tolerated than sulfadoxine–pyrimethamine (González et al., 2018). In the Greater Mekong Subregion efficacy is mixed: a 2011 Cambodian trial reported 19% PCR-corrected artesunate-mefloquine failure (Lek et al., 2022), yet regional cases fell 77% and deaths 97% during 2012–2022 (Manzoni et al., 2024). Pooled adherence to weekly mefloquine reached 80.6% across 16,959 travellers, with weekly regimens outperforming daily ones (Ahluwalia et al., 2020; Rodrigo et al., 2020). Mild adverse reactions affect 32–45% of users (González et al., 2018; Tickell-Painter et al., 2017), but severe neuropsychiatric events occur in only about one per 10,000 travellers (González et al., 2018); anxiety, depression and psychosis underlie FDA-boxed warnings (Ahmad et al., 2021).

Pyrimethamine, an antimalaria drug, is still being used, particularly in combination with sulfadoxine, for chemoprevention in sub-Saharan Africa and other developing regions (Audibert et al., 2025). The sulfadoxine-pyrimethamine combination continues to be part of prophylaxis strategies, particularly for infants and young children under malaria chemoprevention programs (Audibert et al., 2025). Although there have been concerns regarding malaria parasites’ resistance to pyrimethamine, the protective efficacy remains sufficient to justify continued use when the drug is used in combination with sulfadoxine (WHO, 2025).

This study focuses on the potential of utilizing thermosensitive poly (N-acryloyl glycinamide) hydrogel as an antimalarial drug delivery agent for malaria transmission-blocking. Poly (N-acryloyl glycinamide) hydrogel, PNAGA, is a thermosensitive hydrogel that changes from solution to gel with temperature fluctuations. This thermo-responsive characteristic allows PNAGA hydrogels to form a gel upon injection, offering a minimally invasive delivery method and sustained drug release at the desired location (Chen and Hoffman, 1995).

Due to these properties, PNAGA hydrogels have become a versatile platform capable of delivering various therapeutic agents, from small molecules and proteins to nucleic acids (Van Tomme et al., 2005). Their ability to shield encapsulated drugs from degradation, extend the duration of drug release, and provide targeted delivery enables them to be a powerful tool in discovering advanced drug delivery systems (Bromberg and Ron, 1998). As research advances, PNAGA hydrogels offer a potential breakthrough in drug delivery that can significantly enhance treatment outcomes for various diseases.

## 2 Methodology

### 2.1 Materials

Acrylic acid, benzoyl chloride, hydroquinone, mercurochrome solution, glycinamide hydrochloride, and a dialysis tube with a 6/8,000 Dalton cut-off were obtained from Guangdong Wengjiang Chemical Reagent Co., Ltd. (China). Additionally, mercurochrome solution was sourced from Jinbo Marine (China). Diethyl ether, potassium carbonate, charcoal, Celite®, ethanol, methanol, sodium hydroxide (NaOH), sodium chloride (NaCl), phosphate-buffered saline (PBS), hydrochloric acid (HCl), RPMI 1640, and sorbitol were acquired from Sigma-Aldrich®. Blood samples of O+ and AB + types were provided by the National Blood Service Zimbabwe (NBSZ) via the NIHR. Finally, nitrogen (N_2_) and carbon dioxide (CO_2_) were procured from BOC Gases Zimbabwe.

### 2.2 Methodology

#### 2.2.1 Synthesis of acryloyl chloride

The synthesis of acryloyl chloride was performed according to the method outlined by Stempel et al. (1950). A mixture consisting of 216 g (3 mol) of acrylic acid, 844 g (6 mol) of benzoyl chloride, and 0.5 g of hydroquinone was rapidly distilled using an efficient 25-cm distilling column. The distillate was collected in a receiver containing 0.5 g of hydroquinone and was immediately cooled in ice. Throughout most of the distillation process, the temperature at the column’s top ranged between 60 °C and 70 °C, but once it reached 85 °C, the distillation was terminated. The crude product, weighing approximately 215–225 g, was subsequently redistilled using the same column, and the fraction boiling at 72 °C–74 °C at 740 mmHg was carefully isolated (Stempel et al., 1950).

#### 2.2.2 Synthesis of N-acryloyl glycinamide (NAGA)

The synthesis of NAGA followed the method outlined by Boustta et al. (2014). Glycinamide hydrochloride (105 g, 0.951 mol) was placed in a 0.2 dm^3^ three-necked reactor equipped with a mechanical stirrer and ice bath, with nitrogen gas gently flowing through the system. To initiate the process, 10.0 cm^3^ of cool water was added and stirred until the compound was fully dissolved, followed by the sequential introduction of 300 cm^3^ of cool diethyl ether and 560 cm^3^ of cool 2 M potassium carbonate. A solution of 95.1 g (1.05 mol) of freshly distilled acryloyl chloride in 400 cm^3^ of diethyl ether was then added dropwise while maintaining the mixture at 0 °C and stirring at 500 rpm. This reaction was continued for an additional 2 hours at room temperature. The pH was adjusted to two using 6 N HCl, after which the organic layer was separated, and the aqueous phase was washed three times with 300 cm^3^ of diethyl ether. The aqueous solution underwent decolorization using charcoal and was then filtered through Celite®. To remove any residual diethyl ether, the mixture was subjected to a slight vacuum at 20 °C, after which its pH was neutralized using 2 N NaOH. The resulting compound was then freeze-dried, yielding 256 g of a mixture of NAGA and potassium chloride, which was washed three times with 0.15 dm^3^ of a 4:1 ethanol/methanol solution to eliminate organic salts. Following evaporation, 110 g of crude NAGA was obtained. Recrystallization was performed using 1.4 dm^3^ of a 4:1 ethanol/methanol solution, resulting in 78 g of pure NAGA with a melting point of 145 °C and a 64% yield relative to the initial glycinamide (Boustta et al., 2014).

#### 2.2.3 Synthesis of poly (N-acryloyl glycinamide) (PNAGA)

Poly (*N*-acryloyl glycinamide) was synthesized following the method described by Boustta et al. (2014). A precise amount of isopropanol was introduced into a 250 cm^3^ flask, equipped with a magnetic stirrer, containing 5.01 g of NAGA and 10.6 g of potassium persulfate, both dissolved in 78.2 cm^3^ of deionized water. The mixture underwent degassing by bubbling nitrogen gas through it for 1 h. Subsequently, the flask was placed in an oil bath at 60 °C, allowing polymerization to proceed for approximately 48 h. Following this period, the resulting viscous mixture was diluted with 100 cm^3^ of hot water and transferred into a dialysis tube (6/8,000 Dalton cut-off). Upon completion of dialysis against water, the tube contents were carefully collected and freeze-dried, yielding the final PNAGA product (Boustta et al., 2014). The molecular mass of the synthesized PNAGA, *M*_*v*_ = 36,600 g/mol, was determined by viscometry from the intrinsic viscosity $\left[\eta \right]=0.274\;{\text{cm}}^{3}/g$ in water, using the relationship $\left[\eta \right]=1.16\times 10^{-3}\cdot M_{v}^{0.52}$ reported by Haas et al. (1970).

#### 2.2.4 PNAGA-drug formulations

A solution was prepared by dissolving 100 mg of PNAGA in 1 cm^3^ of deionized water and heating it at 70 °C until fully dissolved. Based on previously reported *in vitro* antimalarial assays (Desjardins et al., 1979; Levy, 2020; Moll et al., 2013), scaled to the culture volume used in this study, 0.075 mg of pyrimethamine or 1.25 mg of mefloquine hydrochloride was added, and the suspension was cooled to 37 °C before use. Both formulations were kept at 37 °C until they were introduced into malaria-infected blood for mosquito feeding.

#### 2.2.5 Differential Scanning Calorimetry (DSC) and Thermogravimetric Analysis (TGA)

Differential Scanning Calorimetry (DSC) analysis was performed using the Waters TA Instruments Discovery DSC 25, while Thermogravimetric Analysis (TGA) was carried out using the Waters TA Instruments Discovery TGA 550. For DSC, approximately 5 mg of PNAGA was sealed in an aluminum pan. The sample was equilibrated at −20 °C, heated to 50 °C at 5 °C·min^-1^, cooled to −20 °C at 5 °C·min^-1^, and reheated to 50 °C at 5 °C·min^-1^. The lower start temperature and reduced heating rate were chosen to resolve low-temperature transitions and to avoid thermal degradation observed at higher temperatures. The thermograms were recorded to identify endothermic and exothermic events, including moisture loss, relaxation, and polymer transitions. For TGA, 5 mg of PNAGA was placed in a platinum pan and heated from room temperature to 700 °C at a rate of 10 °C/min under a nitrogen atmosphere. The weight loss profile and derivative thermogravimetric (DTG) curve were analyzed to determine the onset of degradation, major decomposition stages, and residual char content. All measurements were performed in triplicate to ensure reproducibility, and data were processed using TA Universal Analysis software. Prior to sealing, PNAGA samples were dried in a vacuum oven at 60 °C for 24 h to minimize residual moisture, as the polymer is hygroscopic and moisture uptake from ambient air can contribute a significant mass loss signal below 100 °C in TGA and broaden or obscure thermal transitions in DSC.

#### 2.2.6 Drug loading capacity and encapsulation efficiency

Drug encapsulation efficiency and loading capacity of PNAGA formulations were determined using UV-Vis spectrophotometry. PNAGA-drug formulations were prepared by dissolving PNAGA in deionized water and adding either mefloquine hydrochloride or pyrimethamine at 70 °C. The drug-polymer formulations were cooled to 37 °C. To separate the free drug from the polymer-encapsulated drug, the formulations were placed into dialysis tubing with a molecular weight cut-off of 6,000–8,000 Da and dialyzed against deionized water at 37 °C. After equilibrium, aliquots of the dialysate were collected and analyzed using a UV-Vis spectrophotometer at the characteristic absorbance maxima of each drug: 284 nm for mefloquine hydrochloride and 272 nm for pyrimethamine. Calibration curves were constructed for each drug by measuring the absorbance of known concentrations at their respective λ-max values. The concentration of free drug in the dialysate was determined from the calibration curve, and the amount of encapsulated drug was calculated by subtracting the free drug from the total drug initially added (Halayqa and Domańska, 2014; Ramesh et al., 2020; Wang et al., 2021).

To determine the drug load capacity, the following formula was used:

$$DLC\%=\frac{Amount\;of\;drug\;encapsulated}{Total\;weight\;of\;drug+polymer}\;x\;100$$

where *DLC%* is the Percentage Drug Loading Capacity.

To determine how much of the drug was encapsulated, the following formula was used:

$$EE\%=\frac{Amount\;of\;drug\;encapsulated}{Total\;drug\;added}\;x\;100$$

where *EE%* is the Percentage Encapsulation Efficiency.

All measurements were performed in triplicate, and mean values were reported.

#### 2.2.7 Thermal characterization

The sol-gel transition temperature of PNAGA was determined using the reverse test tube method by Boustta and Vert, (2019). Eight milligrams of dried PNAGA was introduced into 8 cm^3^ of de-ionised water in a glass tube, and the mixture was placed in a water bath at 80 °C until total dissolution occurred. The solution was split into two equal portions and cooled down to room temperature. To determine the gel-sol transition temperature, the gel was heated in the solution at the rate of 4 °C/min, and the transition temperature was determined when the gel started to flow along the tube wall. To determine the sol-gel transition temperature, the solution was cooled at a rate of 4 °C/min until gelation occurred. The thermal transition processes were repeated three times (Boustta and Vert, 2019).

#### 2.2.8 Thawing of glycerolyte-frozen *Plasmodium falciparum*

*Plasmodium falciparum* was obtained from the National Institute of Research, Zimbabwe. A vial stored in liquid nitrogen was thawed at 37 °C for 1–2 min, after which the blood sample was transferred to 50 mL centrifuge tubes using a sterile pipette. The blood volume (V) was measured, and 0.1 × V of 12% NaCl was added dropwise while gently shaking the tube. The sample was then left to stand for 5 min. Following this, 10 × V of 1.6% NaCl was slowly added, with continuous swirling of the tube. The mixture underwent centrifugation at 500 *g* at 20 °C for 5 min, and the supernatant was removed. Subsequently, 10 × V of malaria culture media was added dropwise, ensuring gentle mixing before a second centrifugation at 1,500 rpm at 20 °C for 5 min, followed by aspiration of the supernatant. Finally, the pelleted blood cells were resuspended in malaria culture media containing 15% blood serum, and the prepared sample was transferred to a culture flask and a desiccator for further use (Moll et al., 2013).

#### 2.2.9 Blood processing

A sample of complete O+ red blood cells was pipetted into a tube and centrifuged at 1800 RPM for 4 min. The supernatant was aspirated, leaving up to 8 mL of liquid in the tube. RPMI 1640 was then added, and the tube underwent a second centrifugation at 1800 RPM for 4 min, followed by aspiration of the supernatant. The process was repeated once more, with RPMI 1640 added again, and the tube was centrifuged for another 4 min at 1800 RPM (Moll et al., 2013).

#### 2.2.10 Culturing Plasmodium falciparum

The culture medium for *P. falciparum* was prepared by combining RPMI with 12.5% non-immune human AB + serum. A 1:5 ratio of fresh O+ red blood cells (RBCs) and parasitized red blood cells (pRBCs) was then added to 5 mL of the malaria culture medium. The resulting suspension was transferred into a 6-well plate and incubated at 37 °C. The isolates were cultivated under a gas mixture containing 5% O_2_ and 5% CO_2_ in N_2_, ensuring optimal conditions for *P. falciparum* growth (Moll et al., 2013).

#### 2.2.11 Synchronisation of the culture

The parasite culture underwent centrifugation at 600 *g*, forming a pellet from a 4 mL sample containing >5% parasitaemia. Next, 4 mL of 5% sorbitol in distilled water was added and incubated at room temperature for 10 min, with occasional shaking (2–3 times). Following this, the culture was centrifuged again at 600 × g, then washed three times using malaria culture medium and diluted to a 5% haematocrit. Parasitaemia was assessed, and the culture was subcultured as usual. This process was repeated after one cycle (approximately 48 h) to maintain parasite synchrony, with sorbitol treatment conducted weekly (Moll et al., 2013).

#### 2.2.12 Temperature-dependent release of the drug formulations

To investigate temperature-dependent drug release, free drugs (mefloquine and pyrimethamine), polymer–drug formulations (PNAGA-mefloquine hydrochloride [PNAGA-MHQ] and PNAGA–pyrimethamine [PNAGA-PYR]), PNAGA alone, and a negative control were tested. *Plasmodium falciparum* schizonts with an initial parasitaemia of 3.12% were used, with each condition replicated. For comparative efficacy testing, 200 µL of parasite culture was added to each well of a 96-well plate, followed by 50 µL of drug treatment (free drug or PNAGA formulation). Final concentrations were calculated to ensure consistency across conditions. Encapsulation efficiency (EE%) and drug loading capacity (DLC%) were determined (EE%: 93.25% for mefloquine hydrochloride, 95.03% for pyrimethamine; DLC%: 45.17% and 46.67%), providing a quantitative basis for dose selection and enabling comparison between free and encapsulated drugs. Plates were incubated at 37 °C for 24 h, after which parasitaemia was assessed by microscopy. Samples were then transferred to 4 °C for 24 h to induce polymer gelation, and parasitaemia was reassessed to evaluate the effects of temperature on drug release.

#### 2.2.13 Statistical analyses

The sol-gel transition temperature tests were done with three replications. Each release experiment was duplicated. Bar graphs were used to display the means of the different groups. Nonpaired t-test analysis was performed to determine significant differences between the groups. All analyses were conducted using OriginPro 2025 (OriginLab Corporation) software, with significance set at the 5% level.

## 3 Results and discussion

### 3.1 Thermal characterisation of PNAGA

Thermal characterization of PNAGA was carried out using DSC, TGA, and sol–gel transition analysis, providing complementary insights into its amorphous nature, stability limits, and reproducible UCST behavior.

#### 3.1.1 TGA and DSC analysis of PNAGA

**FIGURE 1:**
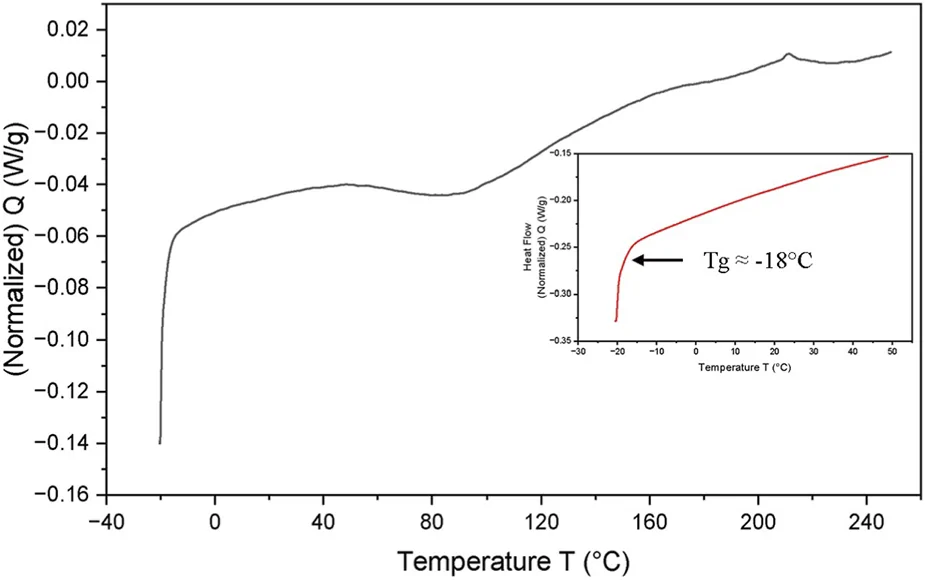
Differential Scanning Calorimetry (DSC) thermogram of dry PNAGA from −20 °C to 250 °C. Insert: short-range scan (−20 °C–50 °C, 5 °C·min^-1^) showing the glass transition (Tg) with midpoint near −18 °C (indicated by arrow), confirming the amorphous nature of the polymer. The broad endothermic signal above 100 °C is attributed to bound water or residual solvent, and the endothermic event near 210 °C is followed by rapid thermal degradation around 220 °C.

Differential Scanning Calorimetry (DSC) provided complementary insights into PNAGA’s thermal behavior across low and extended temperature ranges. The short-range scan (insert, −20 °C–50 °C, 5 °C/min) revealed a reproducible glass transition with a midpoint near −18 °C, confirming the amorphous nature of the polymer and its thermoreversible character as shown in Figure 1. The glass transition midpoint near −18 °C lies well below physiological temperature (37 °C), confirming that PNAGA operates entirely in its rubbery state under all relevant storage and application conditions. In the rubbery state, polymer chain mobility facilitates sol–gel responsiveness upon rehydration, supporting controlled drug release rather than rigid mechanical retention. The absence of melting endotherms in this window further supports the lack of crystalline domains.

Extending the DSC to 250 °C highlighted additional features, including a broad endothermic signal slightly above 100 °C attributed to bound water or residual solvent, and a distinct endothermic event near 210 °C that was immediately followed by rapid degradation around 220 °C. The endothermic event at 210 °C is not assigned as a crystalline melting peak; given the absence of a melting endotherm in the short-range scan and the amorphous character confirmed by the Tg step, this feature is more consistent with the release of enthalpy stored within PNAGA’s dense intermolecular hydrogen-bonding network immediately preceding chain scission. However, since DSC alone cannot fully distinguish a pre-degradation endotherm from a melting event in a partially ordered system, wide-angle X-ray scattering (WAXS) is recommended to independently confirm the absence of crystalline domains. These high-temperature features define PNAGA’s upper thermal stability limit and emphasize that thermal analysis must be performed on rigorously dried samples to avoid confounding contributions from residual water.

**FIGURE 2:**
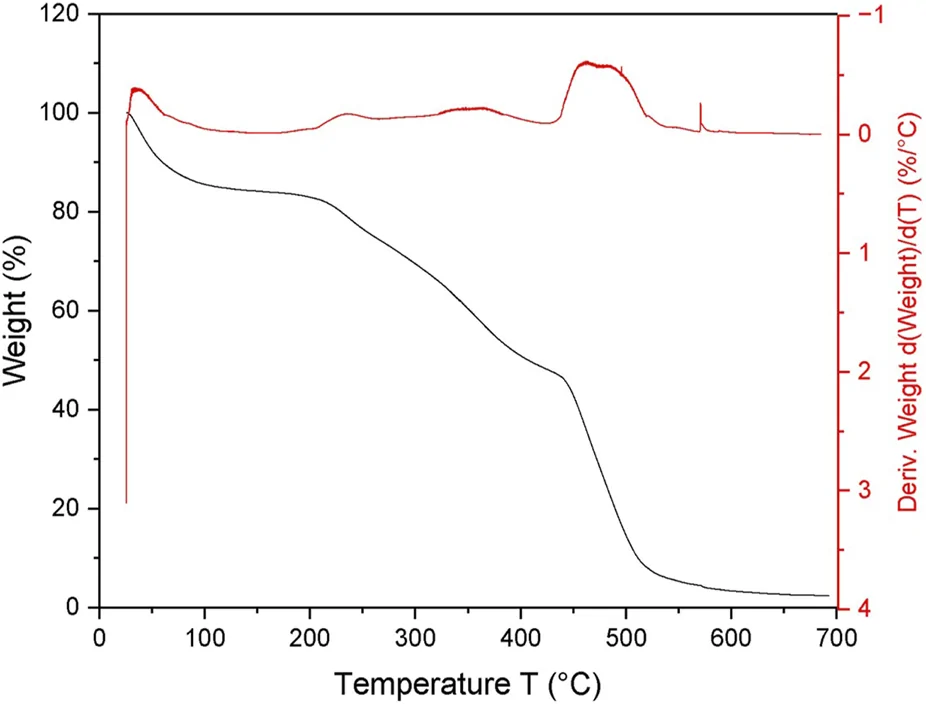
TGA and derivative TGA of PNAGA showing ∼15% mass loss by 100 °C (attributed to bound water/residual solvent) and major decomposition events beginning near 210 °C–220 °C.

Thermogravimetric Analysis (TGA) corroborated these findings (see Figure 2). The TGA profile revealed ∼15% mass loss by 100 °C, indicating that the sample retained a substantial quantity of bound water or residual solvent despite drying. This level of moisture uptake is consistent with PNAGA’s hygroscopic character, arising from its dense amide hydrogen-bonding network, and underscores the necessity of rigorous drying protocols (vacuum oven, 60 °C, 24 h) before thermal characterisation. Major decomposition followed, beginning near 210 °C–220 °C, as confirmed by the derivative TGA peak. The 15% pre-100 °C mass loss precludes claims of broad thermal stability across this region; the more accurate statement is that PNAGA’s polymer backbone remains intact up to ∼210 °C, beyond which irreversible degradation occurs.

The derivative TGA curve confirmed a sharp degradation peak in this region, aligning with the DSC endotherm. Together, DSC and TGA demonstrate that PNAGA is stable across the physiological range but undergoes significant degradation above ∼200 °C.

#### 3.1.2 The sol-gel transition temperatures of PNAGA

For a 10% concentration (w/v), the hydrogel, PNAGA, consistently demonstrated an average sol-gel transition temperature of 22.6 °C ± 1 °C, confirming its thermosensitive behavior. The polymer recorded a transition temperature of 22 °C in the first cycle and 23 °C in both the second and third cycles, demonstrating stability through reproducible transitions across cycles.

The reproducible glass transition and consistent sol–gel transition (∼22.6 °C) establish PNAGA as a reliable thermosensitive hydrogel carrier. Its stability within the biological window ensures controlled drug release at physiological temperatures, while the observed hydration emphasizes the need for drying protocols during formulation. The defined degradation onset provides clear processing limits, ensuring PNAGA’s suitability for biomedical applications where reproducibility and safety are critical. PNAGA’s sol–gel responsiveness within the biological temperature window supports controlled drug release at physiological temperatures. The significant moisture uptake observed in TGA (∼15% by 100 °C) confirms PNAGA’s hygroscopic nature and underscores that drying protocols—specifically vacuum oven treatment at 60 °C for a minimum of 24 h—are essential prior to both thermal characterisation and drug formulation, to ensure reproducibility and accurate interpretation of polymer transitions.

### 3.2 Encapsulation efficiency and drug loading capacity

Both mefloquine hydrochloride and pyrimethamine showed high encapsulation efficiencies of 93.3% and 94.7%, respectively. Drug loading capacities were 1.15% for mefloquine hydrochloride and 0.071% for pyrimethamine. These values place PNAGA at the upper end of reported ranges for polymeric drug delivery systems (Halayqa and Domańska, 2014; Ramesh et al., 2020; Wang et al., 2021) and demonstrate PNAGA’s strong capacity to retain antimalarial drugs within its matrix, ensuring minimal drug wastage; the relatively low DLC% reflects the high polymer-to-drug ratio used. The ability to encapsulate both pyrimethamine and mefloquine hydrochloride suggests PNAGA’s suitability for delivering hydrophobic and amphiphilic antimalarials and combination therapies. High encapsulation efficiency also enables more predictable dosing and improved therapeutic outcomes. Both drugs can interact with PNAGA through hydrogen bonding, van der Waals forces, and hydrophobic interactions due to functional groups such as amines, pyrimidine nitrogens, aromatic rings, and ethyl groups; however, pyrimethamine is smaller and more water-soluble, whereas mefloquine hydrochloride is relatively amphiphilic, which may explain the slight differences observed in DLC and encapsulation efficiency.

### 3.3 Temperature-dependent drug release from PNAGA

#### 3.3.1 Effects of different drug formulations on *Plasmodium falciparum* growth after 24 h at 37 °C

**FIGURE 3:**
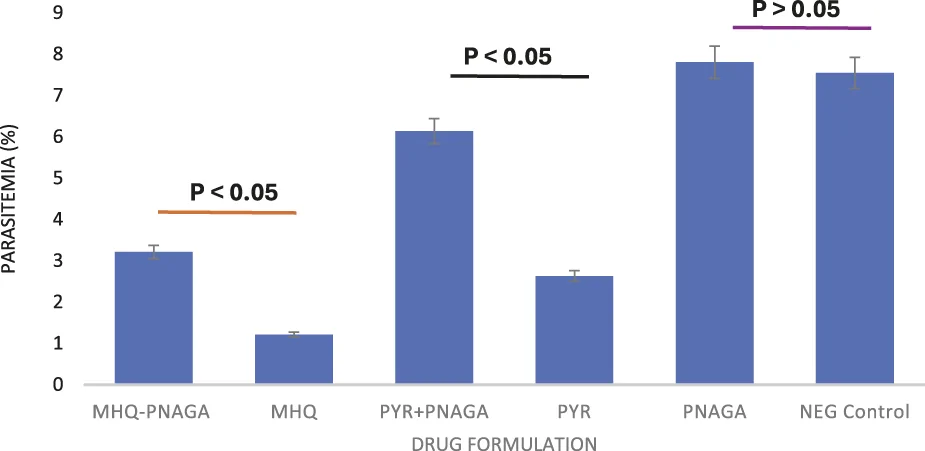
The effects of different drug formulations on *Plasmodium falciparum* growth after 24 h at 37 °C.

Parasitemia assays were used as indirect indicators of drug release, reflecting the availability of encapsulated drugs. In Figure 3, at 37 °C, the polymer-drug combinations had higher parasitemia as compared to the free drugs, pyrimethamine and mefloquine hydrochloride, suggesting that the polymer formulation limits pyrimethamine’s effectiveness in suppressing parasite growth.

The polymer encapsulation reduces drug efficacy by possibly limiting immediate drug availability. PNAGA alone resulted in a parasitemia level similar to the negative control, indicating that it has no intrinsic inhibitory effect on *P. falciparum*. The lack of difference between PNAGA and the negative control suggests that polymer presence alone does not contribute to parasite suppression. The results align with findings from the sol-gel analysis, where the sol-gel transition temperature was 22.67 °C, implying that at a higher temperature of 37 °C, the polymer would be in its solution form.

#### 3.3.2 Effects of different drug formulations on *Plasmodium falciparum* growth after 24 h at 4 °C

**FIGURE 4:**
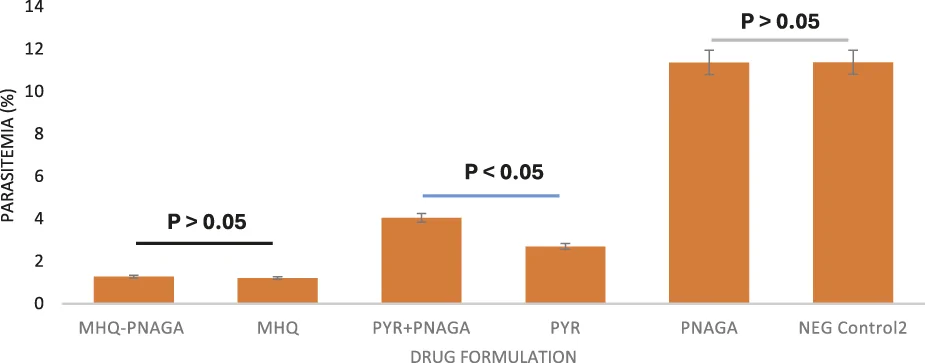
The effects of different drug formulations on *Plasmodium falciparum* growth after 24 h at 4 °C.

Figure 4 shows that at 4 °C, mefloquine hydrochloride (MHQ) alone yielded the lowest parasitaemia, indicating strong parasite suppression.

There was no significant difference between MHQ-PNAGA and free MHQ, indicating comparable effectiveness. In contrast, pyrimethamine (PYR) significantly lowered parasitaemia, confirming its moderate inhibitory effect, while PYR-PNAGA produced higher parasitaemia than free PYR. This suggests that polymer association limits pyrimethamine’s effectiveness by delaying drug availability, likely due to controlled release. PNAGA alone resulted in parasitaemia levels similar to the negative control, confirming that it has no direct anti-parasitic effect and functions purely as a drug carrier rather than an active inhibitor.

#### 3.3.3 Comparison of drug formulation effects on *Plasmodium falciparum* growth at 37 °C and 4 °C

**FIGURE 5:**
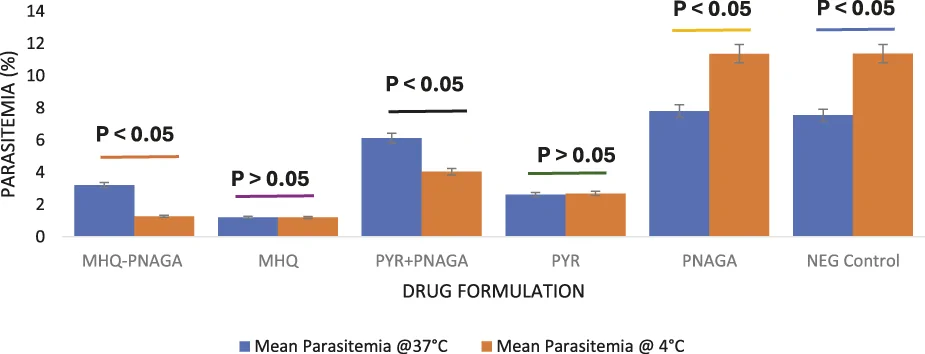
The comparison of the effect of different drug formulations on *Plasmodium falciparum* growth after 24 h at 37 °C and 4 °C.

According to Figure 5, comparisons at 37 °C and 4 °C showed that the free drugs—mefloquine hydrochloride (MHQ) and pyrimethamine (PYR)—produced similar parasitaemia at both temperatures, indicating that their intrinsic efficacy was not temperature dependent. In contrast, the polymer–drug formulations exhibited a marked temperature effect, with higher parasitaemia for MHQ-PNAGA and PYR-PNAGA at 37 °C but lower at 4 °C, consistent with temperature-sensitive release from PNAGA.

This temperature-dependent response aligns with the measured sol–gel transition (∼22.6 °C), where PNAGA remains in the sol state at 37 °C and retains drug, reducing immediate availability, while gelation below the UCST at 4 °C permits greater release for some formulations. PNAGA alone matched the negative control at both temperatures, confirming no intrinsic antiparasitic activity. Overall, these findings demonstrate PNAGA’s UCST-governed, temperature-dependent modulation of drug availability and support its potential for injectable antimalarial delivery, pending further *in vivo* and formulation studies.

## 4 Conclusion

This study confirmed PNAGA as a reproducible, thermosensitive hydrogel with a UCST of 22.6 °C ± 1 °C and thermal stability up to ∼200 °C. The polymer encapsulated mefloquine hydrochloride and pyrimethamine with high efficiency (>93%), demonstrating strong drug-retention capacity. Parasitaemia assays showed free drugs maintained consistent efficacy across temperatures, while PNAGA formulations exhibited temperature-dependent release. At 37 °C, PNAGA retained the drug in its sol state, reducing immediate availability; at 4 °C, gelation enhanced release, especially for MHQ-PNAGA. These findings establish proof of concept for UCST-governed, controlled antimalarial delivery, warranting further *in vivo* and formulation studies. Future work should address *in vivo* pharmacokinetic profiling, cytotoxicity validation, and optimisation of the polymer-to-drug ratio to improve drug loading capacity, alongside exploration of PNAGA formulations for combination antimalarial therapy in transmission-blocking strategies.

## Data Availability

The original contributions presented in the study are included in the article/supplementary material, further inquiries can be directed to the corresponding author.
